# Tropical secondary forests regenerating after shifting cultivation in the Philippines uplands are important carbon sinks

**DOI:** 10.1038/srep22483

**Published:** 2016-03-08

**Authors:** Sharif A. Mukul, John Herbohn, Jennifer Firn

**Affiliations:** 1 Tropical Forestry Group, School of Agriculture and Food Sciences, The University of Queensland, Brisbane, QLD 4072, Australia; 2 Tropical Forests and People Research Centre, University of the Sunshine Coast, Maroochydore DC, QLD 4558, Australia; 3 School of Earth, Environmental and Biological Sciences, Faculty of Science and Engineering, Queensland University of Technology, Brisbane, QLD 4001, Australia

## Abstract

In the tropics, shifting cultivation has long been attributed to large scale forest degradation, and remains a major source of uncertainty in forest carbon accounting. In the Philippines, shifting cultivation, locally known as *kaingin*, is a major land-use in upland areas. We measured the distribution and recovery of aboveground biomass carbon along a fallow gradient in post-*kaingin* secondary forests in an upland area in the Philippines. We found significantly higher carbon in the aboveground total biomass and living woody biomass in old-growth forest, while coarse dead wood biomass carbon was higher in the new fallow sites. For young through to the oldest fallow secondary forests, there was a progressive recovery of biomass carbon evident. Multivariate analysis indicates patch size as an influential factor in explaining the variation in biomass carbon recovery in secondary forests after shifting cultivation. Our study indicates secondary forests after shifting cultivation are substantial carbon sinks and that this capacity to store carbon increases with abandonment age. Large trees contribute most to aboveground biomass. A better understanding of the relative contribution of different biomass sources in aboveground total forest biomass, however, is necessary to fully capture the value of such landscapes from forest management, restoration and conservation perspectives.

Secondary forests comprise more than half of the total forest area in tropical regions and are the dominant forest type^1^. In the tropics, secondary forests also represent a major global carbon sink that rapidly accumulates carbon in aboveground biomass^2^,^3^,^4^. Because they cover a large area in the tropics, accurate estimates of carbon in secondary forests are critical for quantifying the global carbon balance as well as for the successful implementation of climate change mitigation projects^5^. However, there remains a high level of uncertainty in tropical forest carbon accounting, firstly due to the unknown amount of deforestation and forest degradation^6^, and secondly due to a limited number of field studies that have estimated standing biomass in secondary forests^7^. In fact, globally tropical deforestation and forest degradation accounts for approximately 15–35% of anthropogenic carbon emissions^8^, and reversing this trend has a clearly recognized potential for recovering the stocks of forest biomass carbon and for other forest conservation outcomes^9^.

Shifting cultivation or ‘slash-and-burn agriculture’ is a traditional land-use practice in tropical forested landscapes, and is a dominant land-use in rural upland areas in the developing countries^10^. In the tropics, shifting cultivation has also been seen as the primary source of deforestation and forest degradation for many years^11^,^12^. Historically, shifting cultivation has been viewed negatively as contributing to many forms of environmental degradation, including loss of biodiversity and biomass carbon in forests^13^. Accordingly, throughout much of the tropics, governments have developed policies to control or reduce the practice of shifting cultivation by smallholder rural farmers^12^.

In Southeast Asia, secondary forests constitute around 63% of the total forest area^14^, with an estimated 14–34 million people dependent on shifting cultivation for their livelihoods^10^. The extent of land under shifting cultivation however has declined in recent years due to government policies restricting shifting cultivaton and economic factors that promoted other land-use systems^12^,^13^. Consequently, in many parts of this region regenerating secondary forests following shifting cultivation are becoming prominent^10^,^11^,^15^. Due to the dynamic nature of the landscape, shifting cultivation and its changes over time have been very difficult to delineate using satellite based earth observation systems^9^,^15^. In Southeast Asia there is also a lack of spatially explicit knowledge of the forest biomass carbon stocks and carbon dynamics associated with shifting cultivation landscapes. This has limited the inclusion of these landscapes in current negotiations on Reducing Emissions from Deforestation and Forest Degradation (REDD+) in the region to achieve the dual objectives of community development and forest conservation^16^.

In this paper, we report aboveground biomass carbon distribution along a fallow gradient in an upland secondary forest of the Philippines regenerating after shifting cultivation. The Philippines, is both a mega-diverse country and a global biodiversity hotspot, placing it amongst the top priority countries for global conservation^17^,^18^. As in other parts of the developing tropics, shifting cultivation, known as *kaingin* in the Philippines, is a common and controversial land-use in the country^19^. In fact, secondary forests developed after shifting cultivation forms the second largest group of forest in the country after post logging secondary forests^20^. In this paper we also investigate the determinants of biomass carbon recovery in fallow secondary forests, which remain largely overlooked throughout the tropics^4^,^21^. We believe the present study helps address the existing gaps in knowledge on biomass carbon changes and recovery after forest degradation in Southeast Asia which is still largely biased towards the neotropics (see Ngo *et al*.^22^, Saner *et al*.^23^, Kenzo *et al*.^24^ for example). The study is one of the first attempts to systematically assess the carbon in secondary forests associated with slash-and-burn fallows; and will thus substantially improve our understanding of the role that such landscapes play as a sink of atmospheric carbon for both the Philippnes and other tropical developing countries.

## Results

### Distribution of biomass carbon in fallow secondary forests

**Figure 1:**
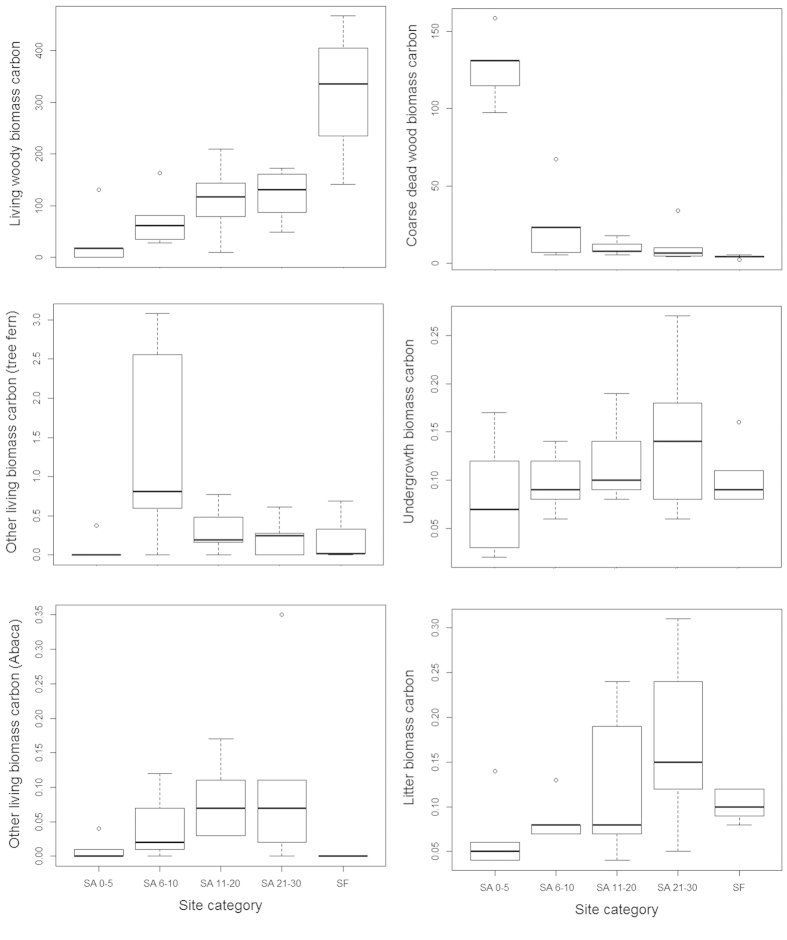
Distribution of aboveground biomass carbon (Mg C ha^−1^) in our study sites on Leyte Island, the Philippines. Each bar indicates upper, lower and median values of biomass carbon allocation, and standard deviation of C allocation under corresponding site category. Note the differences in the Y axis.

**Figure 2:**
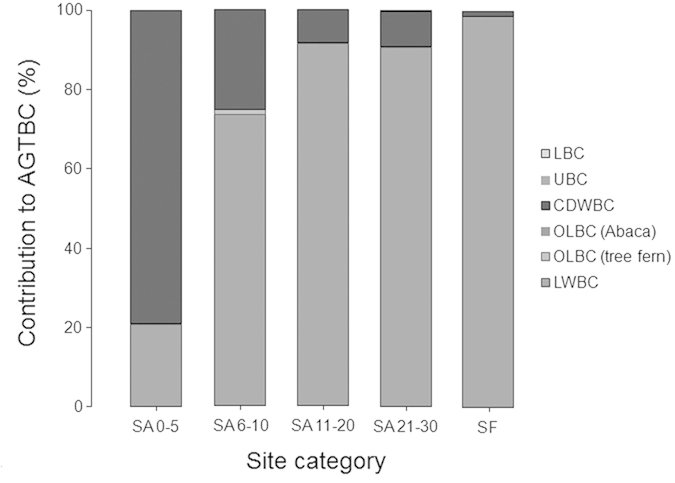
Relative contribution of different source to the total aboveground biomass carbon stock in our sites on Leyte Island, the Philippines.

We measured the biomass of 2918 living tree stems (representing 131 species), 184 tree ferns and 124 Abaca plants (*Musa textilis*, a species of banana native to the Philippines) in our study sites covering a total sample area of 2.5 ha. Tree ferns and Abaca are characteristic species in fallow secondary forests in the area and we include them due to their common occurrence in our study sites. Within our transects we also encountered 1281 pieces of dead woody debris that met the criteria for inclusion for biomass measurement. Using existing allometric models our study thus across all sites finds 328.2 Mg C in the living woody biomass (*LWBC*), 1.18 Mg C in other living biomass (*OLBC;* i.e. tree fern and Abaca), 88.83 Mg C in coarse dead wood biomass (*CDWBC*) and 0.02 Mg C in undergrowth (*UBC*) and litter biomass (*LBC*). Aboveground total biomass carbon (*AGTBC*) was significantly (*F*_*4*_ = 6.07, *p* < 0.01) higher in old-growth forest than the secondary forests of all fallow categories. We found that, carbon in both living woody biomass and coarse dead wood biomass varied significantly (*F*_*4*_ = 9.54, *p* < 0.01) across the sites of different *kaingin* history as expressed by their fallow age (i.e. post *kaingin* period) and in our control old-growth forest (Table 1). There were however no significant differences among the sites when we considered the carbon stored in other living biomass and in undergrowth and litter biomass (Fig. 1). Our post-hoc analysis using Tukey’s HSD revealed significantly higher (321.29 ± 130.96 Mg C; *p* < 0.01) aboveground total biomass carbon in old-growth forest followed by our new (i.e. SA 0–5) and oldest (i.e. SA 21–30) *kaingin* fallow sites. *LWBC* was also significantly higher (316.96 ± 130.63 Mg C; *p* < 0.01) in old-growth forest sites accounting for 98.65% of the *AGTBC*, whilst *CDWBC* was highest (126.65 ± 22.58 Mg C; *p* < 0.01) in our new *kaingin* fallow sites with an estimated 79% contribution to the *AGTBC* (Fig. 2).

**Figure 3:**
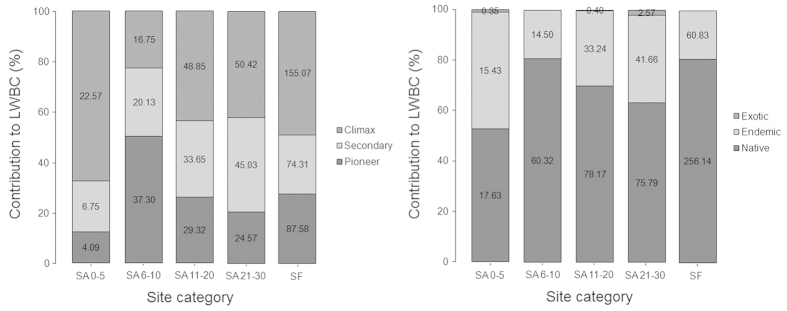
Relative importance of different successional species groups in living woody biomass carbon (*LWBC*) (left); and species of different origin in *LWBC*. Values in the bars indicate absolute contribution (Mg C ha^−1^) to *LWBC* of individual category.

*Parashorea malaanonan* had the highest contribution (33.23%) to the overall *LWBC*, and had relatively greater contribution to all of our fallow sites and old-growth forest. Other than *P. malaanonan, Lithocarpus llanosii, Ficus balete* and *Shorea contorta* contributed respectively 6.39%, 5.32% and 3.89% to the overall *LWBC* (see Supplementary Table 1). In old-growth forest *Calophyllum blancoi* (5.69%), *Petersianthus quadrialatus* (5.26%) and *Bischofia javanica* (4.79%) were other major sources of biomass carbon in living woody stems. When considering species successional guild, climax species were the highest contributers (48.93%; *p* < 0.01) to *LWBC* in old-growth forest sites followed by the oldest *kangin* fallow sites (i.e. SA 21–30) (Fig. 3). Similarly, the contribution of native species to *LWBC* was also significantly higher in the old-growth forest (80.81%; *p* < 0.01) (Fig. 3). As expected, large diameter stems had the greatest contribution to the *LWBC* in our fallow sites, and a significantly high contribution in the old-growth forest (43.28%; *p* < 0.01) (Supplementary Fig. 1). It was a similar case when considering stem heights, where woody stems attaining heights between 30–50 m constituted about 30.11% of the *LWBC*, which was a significantly higher (*p* < 0.01) contribution than other height classes (Supplementary Fig. 2). In the case of *OLBC* as measured for tree fern and Abaca, we found no significant difference in biomass carbon allocation across the sites.

**Figure 4:**
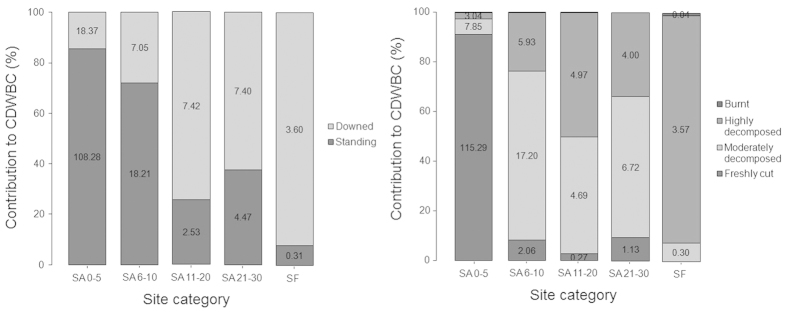
Relative importance of coarse dead wood of different stand form in coarse dead wood biomass carbon (*CDWBC*) (left); and dead woods of different degradation status in *CDWBC* (right). Values in the bars indicate absolute contribution (Mg C ha^−1^) to *CDWBC* of individual category.

In the case of *CDWBC*, carbon stored in standing dead wood was significantly higher (*p* < 0.01) in our new fallow sites contributing about 85.49% to the *CDWBC* (Fig. 4). There were no significant differences in the carbon stored in downed dead wood in our sites of different fallow categories. Post hoc analysis however revealed significantly different (*p* < 0.05) carbon in downed dead wood in new fallow sites and old-growth forest. In new fallow sites the amount of carbon stored in the freshly cut wood was also highest (91.03%; *p* < 0.01), and there were no significant differences in carbon stored in moderately decomposed, highly decomposed and burnt dead wood in different fallow sites and in old-growth forest (Fig. 4). Similarly, we found no significant difference in biomass carbon in litter and undergrowth between our fallow secondary forest sites and in old-growth forest.

### Recovery of biomass carbon in fallow secondary forests

**Figure 5:**
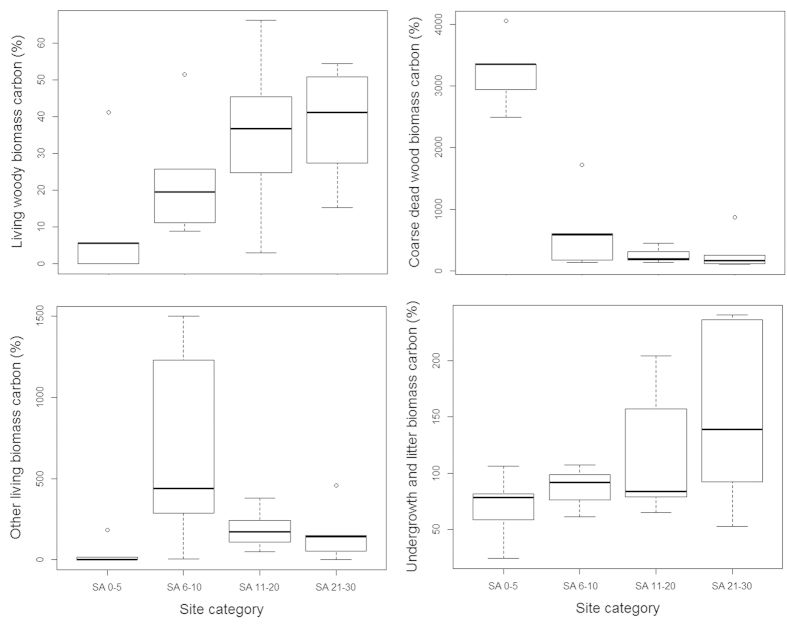
Distribution of aboveground biomass carbon in fallow secondary forest sites in relation to the old-growth forest on Leyte Island, the Philippines. Each bar indicates upper, lower and median values of biomass carbon recovery, and standard deviation of C recovery under corresponding site category. Note the differences in the Y axis.

When compared with old-growth forests used as our control, overall we found that *AGTBC* was highest (49.89 ± 17.39) in the new (i.e. SA 0–5) fallow sites, followed by our oldest fallow sites (41.25 ± 17.76), middle-aged (i.e. SA 11–20) sites (38.10 ± 23.33), and in the young (i.e. SA 6–10) sites (31.46 ± 17.42). The high amount of *AGTBC* in our new fallow sites was mainly driven by the large amount of *CDWBC* remaining in the sites after being cleared and/or used for *kaingin*. Although there was no significant difference, the recovery of *LWBC* was highest in the oldest fallow sites (37.86%), followed by the middle-aged sites (35.28%), young fallow sites (23.4%) and the new fallow sites (10.54%) (Fig. 5). There was no significant difference in the recovery of *OLBC* (in tree fern and Abaca) across our sites, except in the case of new *kaingin* fallow sites and young fallow sites where it was significantly different (*p* < 0.05). The amount of *CDWBC* in relation to that in the control old-growth forest sites was significantly different (*F*_*3*_ = 47.42, *p* < 0.01) across the fallow categories, and was significantly higher in the new *kaingin* fallow sites (i.e. SA 0–5). In fallow secondary forests, woody debris is ultimately lost from the ecosystem with the increasing fallow age but at the same time the regrowth of vegetation offsets the large loss in dead wood in the area. There was however no significant difference in the recovery of undergrowth and litter biomass carbon (*ULBC*) across our fallow sites (of different age categories) compared with the control old-growth forest.

### Factors influencing the recovery of aboveground biomass carbon in fallow secondary forests

Patch size was found to consistently explain the variation in the response variables (i.e. percentage recovery of living woody biomass carbon, *LWBC*; other living biomass carbon, *OLBC*; coarse dead wood biomass carbon, *CDWBC*; and undergrowth and litter biomass carbon, *ULBC*). It also explained the similar amount of variation (models within ∆AIC = 4 are considered equivalent) found in other complex models with more interactions among explanatory variables (Table 2; Table 3). Soil organic carbon, fallow age and the slope of a site were also important in explaining the variation in the recovery of different parameters investigated. Distance from the nearest control forest sites was not retained in any of the best fit candidate models.

## Discussion

### Biomass carbon distribution in tropical fallow secondary forests

We show that secondary forests after shifting cultivation are significant sinks for above-ground carbon. We also show the relatively greater contribution of older fallow areas over young fallow areas as a carbon sink after being used for shifting cultivation in the upland Philippines. In this area, carbon stored in old-growth forests, oldest fallow areas and moderately-aged fallow areas were 321.29 (±130.96) Mg ha^−1^, 132.54 (±57.07) Mg ha^−1^ and 122.41 (±74.95) Mg ha^−1^ respectively, which is comparable to the carbon pools reported from other forests in the Philippines^26^,^27^,^28^. These studies found carbon stored in aboveground biomass ranged between 117.9–305.5 Mg ha^−1^. This estimate is also greater than the upland forests that were selectively logged^27^.

The allometric models and sampling approach used may introduce errors in the estimates of carbon, and thus both may have substantial influences on the results of groundbased forest carbon estimates^7^,^29^,^30^. Locally developed and calibrated allometeric models have the potential to minimize this uncertainty in tropical forest carbon accounting^7^. In our study, we used the most recent allometric model developed by Chave *et al*.^5^ for estimating living woody biomass in tropical forests. Studies on biomass dynamics and forest carbon stocks are biased towards the neotropics, with very limited systematic inventory reported so far from Southeast Asian secondary forests^22^,^23^,^24^. The model developed by Chave *et al*.^5^ is reported to underestimate the aboveground living biomass by 20% when observed biomass exceeded 30 Mg for individual stems, although this trend disappears when a stem’s biomass is between 10–30 Mg^5^. We found that stem density was not the main factor in determining carbon stored in living woody biomass, but diameter and height of individual stems had a better ability to control biomass carbon distribution in forests which is consistent with the observations made by Lasco *et al*.^27^, Rozendaal and Chazdon^31^, Marin-Spiotta *et al*.^32^ and Lawrence^33^ respectively in the Philippines, Costa Rica, Puerto Rico and Indonesia. We also found that, in old-growth forests and in older fallow secondary forests climax species contributed the most in terms of aboveground living woody biomass carbon, which is also in accordance with the finding of Rozendaal and Chazdon^31^. This may reflect the persistence of mature remnant trees in fallow forest when converted from old-growth stands^33^. For example, in all of our fallow sites and old-growth forest *Parashorea malaanonan* consistently made the highest contribution to living woody biomass. In contrast, McNamara *et al*.^34^ have found limited difference in the occurrence of old-growth forest specialist species in secondary forests with different disturbance history in Lao PDR, and argued that it may due to the high resilience capacity of certain species and their quick resprouting ability.

In young *kaingin* fallow areas the ‘other living standing biomass’ (i.e. tree ferns and Abaca in the present study) and coarse dead biomass constitute a major part of the aboveground biomass carbon. This depicts a very different composition in landscape scale total aboveground biomass carbon than that of old-growth forest and older *kaingin* fallow areas. This is largely due to the long disturbance (and use) history and the large remaining amount of dead wood on sites after being used for shifting cultivation^3^. However, Orihuela-Belmonte *et al*.^35^ have found that in Mexico coarse dead wood biomass carbon is higher in older fallow areas and also significantly different across sites of different fallow age. Similar to our findings, Pelletier *et al*.^15^ also reported that aboveground forest biomass carbon is not very different between old-growth forest and older fallow areas, but is different between young fallow areas and old-growth forest.

### Factors influencing the biomass carbon recovery in fallow secondary forests

Recovery of aboveground living tree biomass carbon was highest in the oldest fallow sites and lowest in the new *kaingin* fallow areas, although living tree biomass is the first pool to be affected when forests are converted for shifting cultivation use. Coarse dead biomass carbon was high in the new *kaingin* fallow sites compared to the control old-growth forest site, reflecting the high amount of coarse dead wood in new and relatively young *kaingin* fallow sites remaining after clearing these areas. Other living biomass carbon was highest in the young *kaingin* fallow areas representing the dynamic nature of such landscapes, where undergrowth and litter biomass carbon is found to increase gradually from new to oldest *kaingin* fallow sites. Several studies have found that aboveground biomass carbon recovers rapidly during early successional years after abandonment, followed by a relatively slow recovery rate after reaching a peak or intermediate stage^24^,^33^,^36^,^37^. Such recovery may take place at a rate of between 3.75–9.4 Mg C ha^−1^ year^−1^ and may take as long as 55–95 years^24^,^38^,^39^. In tropical old-growth forests, annual rates of biomass carbon change are typically lower than forests that have been subject to different levels of anthropogenic disturbance^40^, and in such forests the biomass carbon accumulation rates also decrease with an increasing stand age after reaching an intermediate age^31^,^32^.

We found that biomass carbon recovery was constrained mainly by landscape patchiness. In our LMEM patch size showed a consistent control in determining the recovery of biomass carbon at different aboveground levels. In tropical forests, environmental determinants of such recovery as well as its magnitude are still poorly quantified^4^,^21^. Changes in biomass carbon are also driven by the growth and mortality of trees, although such changes are difficult to monitor and require long-term monitoring^41^. It is however clear from our study that aboveground living tree biomass is the most vulnerable carbon pool in tropical secondary forests. A similar observation is also made by Kotto-Same *et al*.^39^. Other environmental factors that also influence the variation in recovery rates are soil organic carbon, fallow age and slope of a site. Distance from the nearest control old-growth forest was found to be unimportant in our LMEM. In the case of other living biomass carbon and coarse dead wood biomass carbon recovery, there was no notable pattern in the LMEM, which may be attributed to the fact that these components have the smallest contribution to our old-growth forest total aboveground biomass carbon.

Chronosequence studies are a widely used approach to investigate secondary forest and successional developments after disturbances^31^,^42^,^43^. Both fallow age and fallow cycles offers an indication of previous forest use^33^, although the present study was limited to only fallow age as the number of cycles was unknown. We found that recovery of standing living woody biomass carbon was distinct across sites of different categories and was superior in older *kaingin* fallow sites. In young fallow areas, although the number of stumps was higher, biomass carbon recovery was higher in sites with larger diameter trees as also mentioned by Rozendaal and Chazdon^31^. Many environmental factors influence secondary forest recovery after disturbances^11^,^44^, and studies have demonstrated different recovery rates depending on the site’s geographic position together with biotic and abiotic attributes^11^,^38^,^45^.

Biomass accumulation specifies the carbon stored in aboveground biomass, and was reported to be declining by 9.3% with each fallow cycle in Indonesia^11^,^33^,^46^. This decline was mainly driven by the density and biomass of woody stems >10 cm dbh as well as soil phosphorus availability^33^. Burning also has a positive influence on biomass carbon accumulation in fallow secondary forests^24^,^47^. Recovery may also depend on remaining forest cover in a landscape, although intensity of past land-use rather than edaphic variables is the strongest predictor of biomass recovery^48^. Rapid biomass recovery during secondary forest succession was also reported by Letcher and Chazdon^37^ and Martin *et al*.^44^.

### Forest management and landscape restoration implications

In tropical forests, aboveground biomass carbon dynamics are important in net primary productivity, and regardless their large contribution to the global carbon balance, uncertainty yet remains regarding their quantitative contribution to the atmospheric carbon cycle^7^,^49^. In Southeast Asia there are large areas of secondary forest as a result of past anthropogenic disturbances. The existing carbon measurement uncertainties create critical data gaps that limit our understanding of the important role of these forests as sources and sinks of terrestrial carbon^16^. In recent years, it is also increasingly recognized that although undervalued, tropical secondary forests can provide the same important ecosystem goods and services as primary or old-growth forests^32^,^50^,^51^. In tropical regions deforestation has been a large contributor of greenhouse gas emissions, and reversing these trends with suitable land-use(s) has a clearly recognized potential for recovering biomass carbon stock in forests^9^,^52^. Compared to other climate mitigation options, regenerating secondary forests offers a low-cost approach to reducing greenhouse gas emissions in the tropics, although a greater understanding of and capability to quantify carbon dynamics in such novel and emerging ecosystems is necessary at landscape scales^50^. For instance, in many tropical countries monocultures have been preferred for reforestation, but our study along with others suggest that considerable net primary productivity and carbon storage could be achieved if more species diversity is secured in tropical landscapes^53^. Current remote sensing based techniques using satellite imagery offer promise for estimation of ecosystem carbon exchange in complex forested landscapes, although large variability exists depending on forest conditions and landscape type^54^,^55^. A combination of field-based inventory and remote sensing techniques can be used to reduce such variability and to cover large areas of tropical forests^15^.

## Conclusion

Our results highlight that the secondary forests regenerating following shifting cultivation are important carbon sinks in tropical ecosystems. Allowing development of such secondary regrowth has clear potential for carbon storage in the aboveground forest biomass. Biomass carbon distribution differs across sites with different land-use histories, and a large amount of carbon is stored in living woody biomass in older fallow areas indicating the dynamic nature of the landscape and succesional development towards undisturbed forests. In young fallow areas, large amounts of carbon are stored in coarse dead wood material which ultimately provided inputs to the soil for biomass accumulation in living trees. We found that patch size is an important factor in biomass carbon recovery together with soil organic carbon, fallow age and the slope of a site, and after thirty years a site may achieve more than 40% of the biomass carbon found in old-growth forest without any history of major disturbances. The extensive deforestation and forest degradation in tropical regions caused by shifting cultivation and other land-uses is being blamed for biodiversity loss and global warming. We found that regenerating secondary forests have the potential to mitigate the impacts of such deforestation and forest degradation and to contribute to global carbon sequestration. However, it remains necessary to determine exactly where in the aboveground forest biomass the carbon is being sequestered (e.g. in the present study, we found that coarse woody debris in new *kaingin* fallow sites has the highest contribution to aboveground total biomass carbon, and in oldest *kaingin* fallow sites living woody biomass carbon had the highest contribution).

## Methods

### Study area

**Figure 6:**
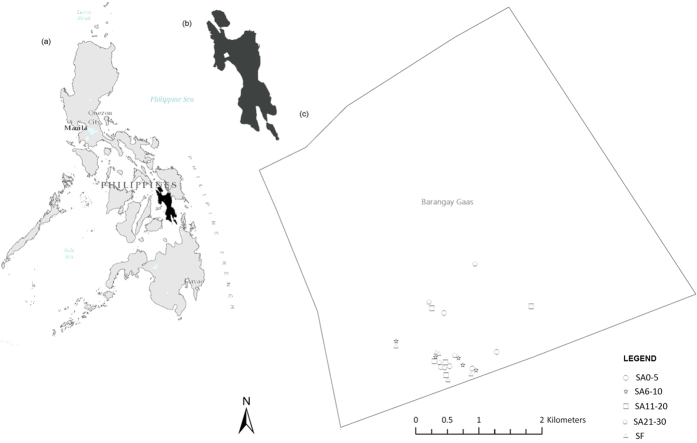
Map of the Philippines (a), with location map of Barangay Gaas on Leyte Island (b) and our study sites in Gaas (c). Spatial position of the site locations were plotted in global geo-political boundary available from Esri (http://www.arcgis.com/) using ArcMap (version 10.3) software.

The study was conducted in Barangay (the smallest administrative unit in the Philippines and native Filipino term for village) Gaas on Leyte Island, the Philippines (Fig. 6). Leyte is the eighth largest island in the Philippines, and our study site was located on the western side of the island. Geographically, Leyte is located between 124°17′ and 125°18′ East longitude and between 9°55′ and 11°48′ North latitude, and covers an area of about 800,000 ha. Forest cover on the island is about 10%, although the once dipterocarp-rich rainforests now comprise mainly patches of old-growth and primary forests, and coconut (*Cocos nucifera*) and Abaca plantations^56^. The relatively flat lowlands of the island are being used for agricultural crop production, especially rice (*Oryza sativa*) and corn (*Zea mays*)^56^.

Leyte Island was formed from tectonic movement and plate convergence which started during the tertiary and quaternary age^56^,^57^. Based on the Coronas Classification of Climate, Leyte has a ‘type IV’ climate with two distinct season^58^. The area enjoys a relatively even distribution of rainfall throughout the year with annual rainfall totalling approximately 4,000 mm^59^. Mean annual temperature is 28 °C which remains constant throughout the year^58^. Relative humidity ranges between 75 to 80 percent during the dry and the wettest months^60^. The soil in our study area in Gaas was an Andisol type which possesses a markedly higher soil organic carbon content than rest of the islands^58^.

### Site selection

We chose Barangy Gaas (also refer to as Gaas) purposively. This area of Leyte is situated in a comparatively high altitudinal range and compared to other parts of the island it has a relatively greater extent of undisturbed forests. It also has a low population density. These critieria are prerequisites for the *kaingin* fallow to regrow as secondary forests^61^. For our study we consider only the *dahilig kaingin* system which is identical to the most common practice of shifting cultivation in the tropics (see Olofson^62^ for details of the Philippines *kaingin* systems). Smallholder farmers living in the area usually grow Abaca or coconut in their fallow *kaingin* area in order to receive financial benefits during the time of abandonment. Our study was however confined to the areas where farmers cultivated only Abaca since coconut plantations generally involve more intensified land-management during the fallow periods and this is not conducive for secondary forest development.

### Biomass Inventory

A series of extensive field surveys were undertaken at the sites between May and October 2013. For the biomass inventory we followed a modified Gentry plot approach^63^. This method has been reported as the most efficient for monitoring secondary forest development in tropical regions^64^. We categorized our sites into four different fallow categories; i.e. less than 5 year old fallow (SA0–5), also referred to as new, 6–10 year old fallow (SA6–10) also referred to as young, 11–20 year old fallow (SA11–20) also referred to as middle-aged, and 21–30 year old fallow (SA21–30) also refered to as oldest. We limited our study to fallow forest sites that were at least 1 ha in size^45^. Additionally, we sampled old-growth forest (SF) as control forest sites. These forests had no history of *kaingin* and logging and were located close to our fallow sites. Our control forest sites were structurally and floristically similar to primary forests although they may have undergone a limited level of anthropogenic use (e.g. source of firewood, wild fruits etc.) like most of the forest in the tropical forest-agriculture frontiers.

We identified a total of 25 sites (four fallow categories +old-growth forest x five replicates). Both the fallow age and fallow cycles have been reported to influence the biomass dynamics in secondary forests^3^. In our study we were only able to consider the fallow age and not the fallow cycles due to a lack of reliable information about past site history. At each site, four transects of 50 m × 5 m were established parallel to each other and with a minimum of 5 m distance between transects, representing a total area of 0.1 ha per site. For standing live trees and palms ≥5 cm diameter at breast height (dbh) we recorded diameter and height of each individuals at 1.3 m from the ground or above stem abnormalities (e.g. buttresses, stilt roots etc.). All individuals were identified to the species level and named with the help of a local expert from Visyas State University (VSU). In the case of unknown species we used the most common Filipino name for that species. We also measured all tree ferns and Abaca ≥5 cm dbh in our transects as other living biomass because they represent a major component of secondary forest succession in post-*kaingin* secondary forests in the Philippines^19^. Lianas were not included in our study. For measuring the dbh of individual tree stems we used diameter tapes. We used a hypsometer for tree height measurements, although the closely-structured canopy in tropical forests sometimes made it difficult to measure tree heights with a high reliability.

Since slashing and burning is a common practice in shifting cultivation landscapes, coarse dead wood biomass in the form of felled, degraded and burnt trees comprise a significant part of the total aboveground biomass in such areas^10^,^15^. Consequently we censused all dead, cut and burnt trees ≥5 cm at dbh that fell within in our transects. For each individual stem, we recorded whether it was standing or downed (i.e. fallen), and the degradation staus, categorized as – freshly cut, moderately decomposed, highly decomposed and burnt^23^. For litter and undergrowth (i.e. seedlings, saplings, shrubs and herbaceous plants) we followed a destructive sampling approach. A 1 m × 1 m rectangular plot was laid in the centre of each of our 100 transects distributed in 25 sites, and all litter and undergrowth samples were collected and weighed in the plot using a measuring balance.

Additionally, for each site we recorded the site geographic position, elevation (*E*), distance from the nearest control forest (*D*), patch size (*PS*), slope (*SL*), leaf area index (*LAI*) and soil organic carbon (*SOC*) as a percentage. We used a digital plant canopy imager (Model: CID Bio-Science, CI-110/120) for measuring leaf area index and a hand-held global positioning system (Model: Garmin eTrex) for elevation.

### Biomass calculation in forest ecosystems

There is no allometric equation which is specifically developed for the secondary forests in the Philippines^28^. Consequently we used the generic allometric model develeoped by Chave *et al*.^5^.


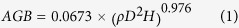


where *AGB* or aboveground dry biomass is in kg, *D* is the dbh in cm, *H* is the height of the tree and/or palm in m and *ρ* is the species specific wood density (g cm^−3^). This model performed better than the widely accepted previous model by Chave *et al*.^7^, and performed well across all forest types and bioclimatic conditions^5^. The inclusion of tree height in this model provides more reliable estimates of biomass, compared to the pervious models that used only diameter in the model^5^,^65^. Moreover, this model is based on 58 global sites distributed across the tropics where the previous model was based on 27 global sites^5^,^7^. For species specific wood density we used the World Agroforestry Centre’s wood density database where wood density was the ratio of dry mass to the green volume^66^ (Supplementary Table 3). In the case of unknown species or where wood density was not available, we took the mean wood density of the genus as a substitute^23^.

For tree ferns and Abaca we used the following allometric models developed by Stanley *et al*.^67^ and Armecin and Coseco^68^ respectively.


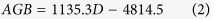



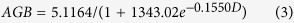


where *AGB* for ferns and abaca is respectively in g and kg, and *D* is the diameter of individuals in cm.

The volume of coarse woody debris per area was calculated from transect data, and we used the following equation to obtain the volume of individual stems that fell within or intersected our transects.


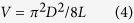


where *V* is the volume per stem, *L* is the total length of the stem (of coarse woody debris) that fell within or intersected our transects in m and *D* is the diameter of coarse woody debris. Wood density was determined locally by the water displacement method taking representative samples (n = 5) for each of the four wood degradation status (i.e. freshly cut, moderately decomeposed, highly decomposed and burnt), and were 0.48 g cm^−3^, 0.35 g cm^−3^, 0.25 g cm^−3^ and 0.19 g cm^−3^ respectively for our freshly cut, moderately decomposed, highly decomposed and burnt wood samples^69^. All biomass measurements were first made at the site level (Mg), and then converted to a per hectare (ha) value after correcting plot size or transect length for the slope^15^.

### Estimating carbon in aboveground forest biomass

In our study total aboveground biomass carbon (*AGTBC*) is the sum of aboveground living woody (tree and palms) biomass carbon (*LWBC*), other living biomass carbon (*OLBC*) measured for tree ferns and Abaca, coarse dead wood biomass carbon (*CDWBC*), undergrowth biomass carbon (*UBC*), and litter biomass carbon (*LBC*). To convert the aboveground biomass of trees we assumed that 50% of the dry mass was carbon^30^. Studies at nearby sites with similar forest types have found average carbon content close to 50%^23^,^24^,^28^. For tree ferns and Abaca, carbon content was assumed to be 50% and 47.3% respectively by total dry biomass after Armecin and Coseco^68^ and Beets *et al*.^70^.

Measuring of the dry biomass litter and undergrowth samples was undertaken in the VSU-ACIAR Analytical Chemistry Laboratory. The samples were air dried at room temperature and then grinded using an electric grinder. The samples were then oven dried at 70 °C for 24 hours and weighed. A representative subsample from each of the litter and undergrowth samples was analysed for estimating the carbon content (% C-Heanes) (Supplementary Table 4). In the case of coarse woody debris we took 5 subsamples from each of the degradation states as mentioned earlier, and the carbon content was measured locally in the laboratory.

### Estimating the recovery of biomass carbon in forests

We compared the recovery of biomass carbon in different aboveground components with that of the control old-growth forests. We combined both tree fern and Abaca as other living biomass carbon (*OLBC*) during the analysis due to their relatively low contribution to *AGTBC*. Recovery (*R*) was expressed as a percentage (%) of biomass carbon using the following equation.


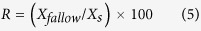


where *X* fallow is the measure of biomass carbon in a fallow site, and 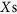 is the mean of corresponding biomass carbon in a similar ecosystem in the control old-growth forest.

### Statistical analysis

We performed both the analysis of variance (ANOVA) and Tukey’s post-hoc test to test any significant difference between the variables. We developed linear mixed-effect models (also refered to as LMEM) to examine the effect of fallow age and selected site attributes (see Supplementary Table 2) on recovery of biomass carbon, using the package ‘nlme’. In our LMEM, fallow age (*FA*), slope (*SL*), distance from the nearest control forest (*DIS*), patch size (*PS*), leaf area index (*LAI*) and soil organic carbon (*SOC*) were used as explanatory variables (i.e. fixed factors), and biomass carbon in different forest strata was the response variable. We used sites nested in fallow categories as the random effect in our models. Due to their high collinearity with other explanatory variables, ‘elevation’ and ‘*LAI’* were excluded from the final LMEM (Supplementary Table 5). All analyses were performed using ‘R’ Statistical package (version 3.0.1). We considered Akaike Information Criterion corrected for small sample sizes (AICc) for the selection of our top models, where the best models had the lowest AICc scores. We used the R-package ‘MuMin’ for our model selection, and to evaluate the contribution different fixed effects had on explaining the variabiont in the response variables^25^. We considered models within four AICc units to be equivalent models^71^.

## Additional Information

**How to cite this article**: Mukul, S. A. *et al*. Tropical secondary forests regenerating after shifting cultivation in the Philippines uplands are important carbon sinks. *Sci. Rep.* **6**, 22483; doi: 10.1038/srep22483 (2016).

## Supplementary Material

Supplementary Information

## Figures and Tables

**Table 1:** Summary (mean ± SE) of aboveground biomass carbon (Mg C ha^−1^) distribution in our study sites on Leyte Island, the Philippines.

Parameter	Fallow category				Old-growth forest
	≤5 year	6–10 year	11–20 year	21–30 year	
***LWBC***	33.42 (±55.25)	74.18 (±54.15)	111.81 (±74.65)	120.02 (±52.05)	316.96 (±130.63)*
Pioneer	4.09 (±5.31)	37.30 (±43.85)	29.32 (±20.78)	24.57 (±6.54)	87.58 (±45.63)
Secondary	6.75 (±7.97)	20.13 (±6.28)	33.65 (±22.4)	45.03 (±18.78)	74.31 (±36.66)
Climax	22.57 (±49.0)	16.75 (±12.27)	48.85 (±55.56)	50.42 (±35.68)	155.07 (±84.03)*
Native	17.63 (±23.35)	60.32 (±42.23)	78.17 (±52.47)	75.79 (±33.68)	256.14 (±108.27)*
Endemic	15.43 (±32.71)	14.50 (±12.03)	33.24 (±28.13)	41.66 (±30.67)	60.83 (±31.7)
Exotic	0.35 (±0.79)	0	0.4 (±0.61)	2.57 (±5.14)	0
***OLBC***	0.09 (±0.17)	1.45 (±1.34)	0.40 (±0.27)	0.34 (±0.37)	0.21 (±0.30)
Tree fern	0.08 (±0.17)	1.41 (±1.33)	0.32 (±0.31)	0.23 (±0.25)	0.21 (±0.30)
Abaca	0.01 (±0.02)	0.04 (±0.05)	0.08 (±0.06)	0.11 (±0.14)	0
***CDWBC***	126.65 (±22.58)*	25.26 (±25.0)	9.95 (±4.97)	11.87 (±12.57)	3.91 (±1.17)
Standing	108.28 (±19.46)*	18.21 (±24.74)	2.53 (±1.07)	4.47 (±5.74)	0.31 (±0.25)
Downed	18.37 (±11.70)	7.05 (±5.68)	7.42 (±5.49)	7.40 (±7.33)	3.60 (±1.07)
Freshly cut	115.29 (±22.2)*	2.06 (±2.77)	0.27 (±0.28)	1.13 (±1.85)	0
Moderately degraded	7.85 (±7.69)	17.20 (±25.39)	4.69 (±3.68)	6.72 (±8.45)	0.3 (±0.21)
Highly degraded	3.04 (±3.56)	5.93 (±4.47)	4.97 (±3.76)	4.0 (±4.5)	3.57 (±1.23)
Burnt	0.47 (±1.01)	0.08 (±0.13)	0.02 (±0.05)	0.01 (±0.02)	0.04 (±0.09)
***UBC***	0.08 (±0.06)	0.10 (±0.03)	0.12 (±0.04)	0.15 (±0.08)	0.11 (±0.04)
***LBC***	0.07 (±0.04)	0.09 (±0.03)	0.13 (±0.09)	0.17 (±0.10)	0.10 (±0.02)
***AGTBC***	**160.3 (±55.86)**	**101.09 (±55.98)**	**122.41 (±74.95)**	**132.54 (±57.07)**	321.29±130.96)*

Where, *LWBC* = living woody biomass carbon, *OLBC* = other living biomass carbon, *CDWBC* = coarse dead wood biomass carbon, *UBC* = undergrowth biomass carbon, *LBC* = litter biomass carbon, *AGTBC* = aboveground total biomass carbon. ^*^Values are significantly different at *p* < 0.01 level.

**Table 2:** Summary of LMEM between site biomass recovery with environmental attributes obtained using the package MuMin^25^.

Parameter	Explanatory variable					*DF*	*LL*	*AICc*	*∆ AICc*	*Weight*
	*FA*	*DIS*	*SL*	*PS*	*SOC*					
*LWBC*				X	X	6	−**68.93**	**157.51**	**0.00**	**0.31**
				X		5	−71.81	158.62	1.12	0.18
			X	X		6	−69.88	159.40	1.90	0.12
	X			X	X	7	−67.14	159.48	1.98	0.11
	X		X	X		7	−67.50	160.20	2.70	0.08
			X	X	X	7	−67.52	160.25	2.74	0.08
	X			X		6	−70.34	160.31	2.81	0.08
					X	5	−73.04	161.07	3.57	0.05
*OLBC*	X		X	X	X	8	−**121.09**	**271.28**	**0.00**	**0.47**
	X			X	X	7	−124.55	272.44	1.16	0.27
			X	X	X	7	−124.90	273.12	1.85	0.19
				X	X	6	−128.31	275.08	3.80	0.07
*CDWBC*	X		X	X	X	8	−**127.61**	**284.31**	**0.00**	**0.76**
	X		X		X	7	−131.62	286.57	2.26	0.24
*ULBC*	X			X	X	7	−**92.14**	**207.62**	**0.00**	**0.55**
				X	X	6	−95.83	210.13	2.51	0.16
	X		X	X	X	8	−90.61	210.32	2.70	0.14
			X	X	X	7	−94.08	211.50	3.89	0.08
	X			X		6	−96.57	211.60	3.98	0.07

Where, *LWBC* = living woody biomass carbon, *OLBC* = other living biomass carbon, *CDWBC* = coarse dead wood biomass carbon, *ULBC* = undergrowth and litter biomass carbon, *FA* = fallow age, *DIS* = distance (from the nearest control forest site), *SL* = slope, *PS* = patch size, *SOC* = soil organic carbon.

^*^DF— Degree of Freedom, LL— Log Likelihood, AIC—Akaike Information Criterion corrected for small sample size. **Values in the bold indicate the most influential model describing the variation in biomass carbon recovery.

**Table 3:** The relative importance of site environmental attributes in the final LMEM.

Parameter	Explanatory variable*					Number ofmodels
	*FA*	*DIS*	*SL*	*PS*	*SOC*	
*LWBC*	0.27 (3)	—	0.28 (3)	0.95 (7)	0.55 (4)	8
*OLBC*	0.74 (2)	—	0.66 (2)	1.0 (4)	1.0 (4)	4
*CDWBC*	1.0 (2)	—	0.76 (1)	1.0 (2)	1.0 (2)	2
*ULBC*	0.77 (3)	—	0.22 (2)	1.0 (5)	0.93 (4)	5

Where, *LWBC* = living woody biomass carbon, *OLBC* = other living biomass carbon, *CDWBC* = coarse dead wood biomass carbon, *ULBC* = undergrowth and litter biomass carbon, *FA* = fallow age, *DIS* = distance (from the nearest control forest site), *SL* = slope, *PS* = patch size, *SOC* = soil organic carbon.

^*^Values in the parenthesis indicate the number of models containing respective explanatory variable.
