# Supplementary material for: Tropical secondary forests regenerating after shifting cultivation in the Philippines uplands are important carbon sinks
Source: Sci Rep. 2016 Mar 8;6:22483. doi: 10.1038/srep22483 (PMC4782068; doi:10.1038/srep22483)
Supplement: Supplementary Information [file srep22483-s1.pdf]

SUPPLEMENTARY MATERIALS

Tropical secondary forests regenerating after shifting cultivation in the Philippines uplands are important carbon sinks

Sharif A. Mukul[1,2*], John Herbohn[1,2], Jennifer Firn[3]

[1]*Tropical Forestry Group, School of Agriculture and Food Sciences, The University of Queensland, Brisbane, QLD 4072, Australia*
[2]*Tropical Forests and People Research Centre, University of the Sunshine Coast, Maroochydore DC, QLD 4558, Australia*
[3]*School of Earth, Environmental and Biological Sciences, Faculty of Science and Engineering, Queensland University of Technology, Brisbane, QLD 4001, Australia*

*Corresponding author: s.mukul@uq.edu.au / sharif_a_mukul@yahoo.com

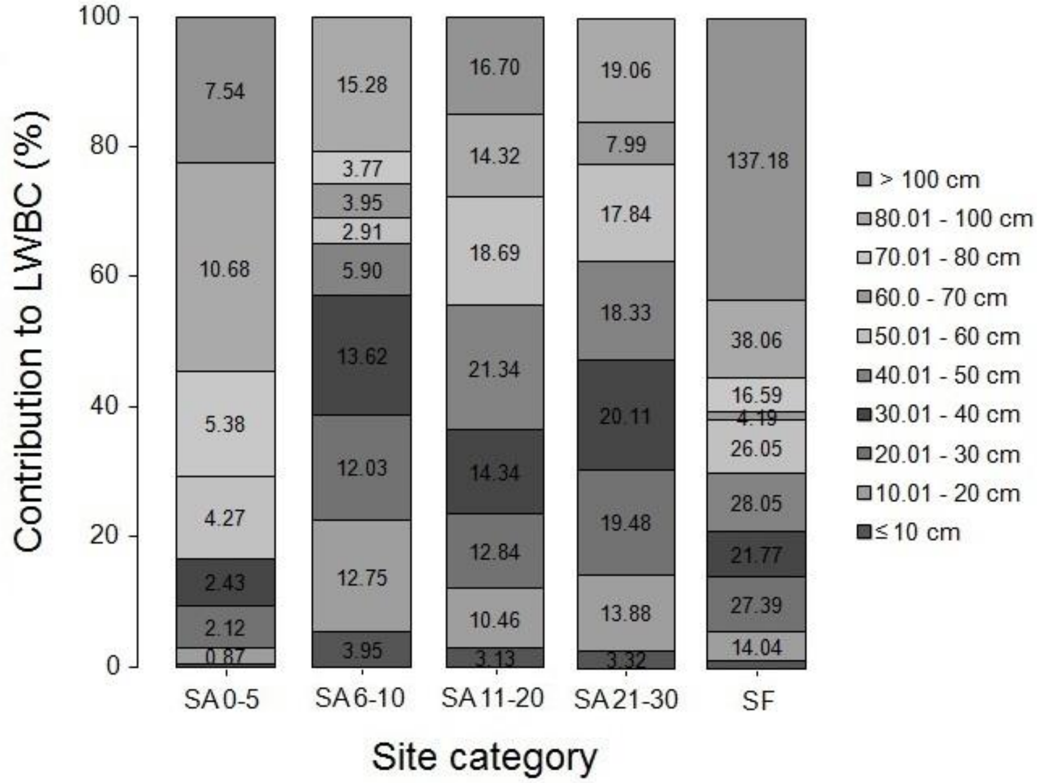

Supplementary Figure 1. Relative contribution of living stems of different diameter class in living woody biomass cabon (*LWBC*); values in the bars indicate absolute contribution (Mg C ha $^{-1}$) to *LWBC* of respective diameter class.

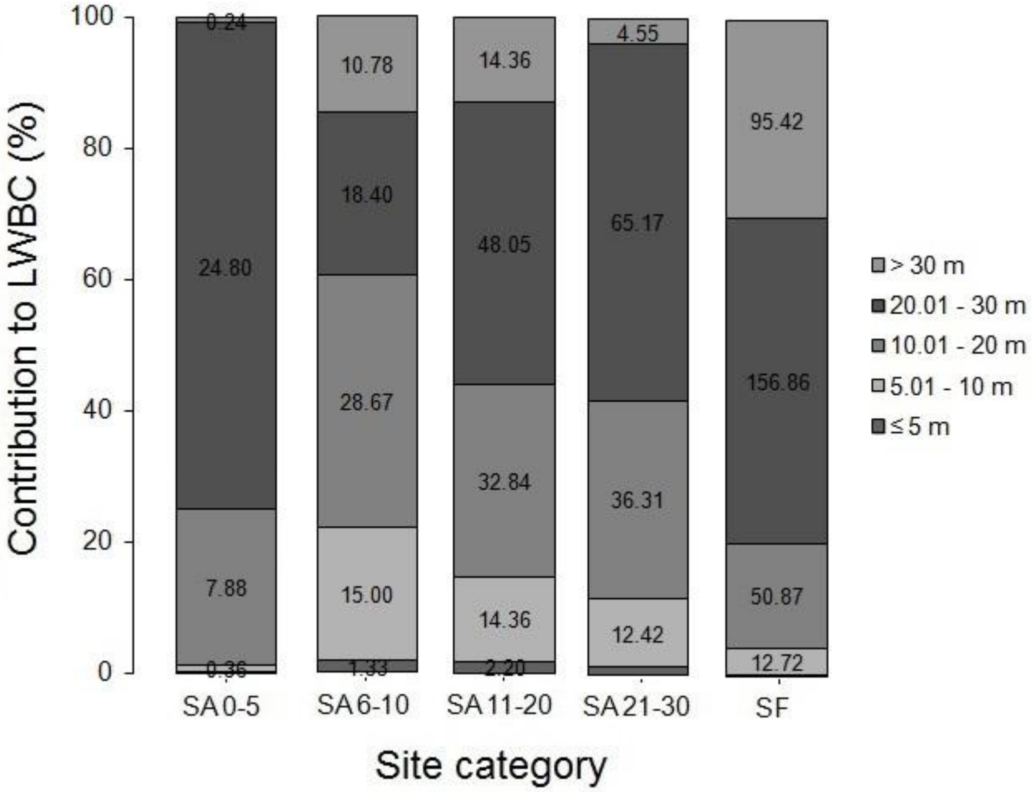

Supplementary Figure 2. Relative contribution of living stems of different height class in living woody biomass carbon (*LWBC*); values in the bars indicate absolute contribution (Mg C ha $^{-1}$) to *LWBC* of individual height class.

Supplementary Table 1. Biomass carbon (Mg C) distribution in living woody species (*LWBC*) in our study sites on Leyte Island, the Philippines.

Botanical name	All sites	Site category				
		SA0-5	SA6-10	SA11-20	SA21-30	SF
Alangium javanicum (Bl.) Wang	6.21 (1.95%)*	0 (0)	0.91 (2.45%)	0.97 (1.74%)	2.9 (4.83%)	1.43 (0.90%)
Albizia falcataria (L.) Fosberg.	0.08 (0.03%)	0 (0)	0 (0)	0.01(0.02%)	0.07 (0.1%2)	0 (0)
Albizia saponaria(Lour.) Blume ex Miq.	0.45 (0.14%)	0 (0)	0.02 (0.05%)	0 (0)	0.02 (0.04%)	0.41 (0.26%)
Alstonia macrophylla G. Don	0.15 (0.05%)	0 (0)	0.03 (0.07%)	0.07 (0.13%)	0.05 (0.08%)	0 (0)
Alstonia parvifolia Merr.	0.01 (0)	0 (0)	0 (0)	0.01 (0.01%)	0 (0)	0 (0)
Anisoptera thurifera	0.01 (0)	0 (0)	0 (0)	0 (0)	0.01 (0.02%)	0 (0)
Antidesma ghaesembilla Gaertn.	1.63 (0.51%)	0 (0)	1.17 (3.16%)	0 (0)	0.29 (0.48%)	0.17 (0.11%)
Ardisia pyramidalis (Cav.) Pers. ex A. DC.	1.60 (0.50%)	0 (0)	0.65 (1.76%)	0.27 (0.49%)	0.09 (0.15%)	0.58 (0.37%)
Areca cathecu L	0.15 (0.05%)	0 (0)	0 (0)	0.06 (0.11%)	0.09 (0.15%)	0 (0)
Arthrophyllum cenabrei Merr.	0.09 (0.03%)	0 (0)	0 (0)	0.09 (0.16%)	0 (0)	0 (0)
Artocarpus blancoi (Elmer) Merr.	2.09 (0.65%)	0 (0.02%)	0.18 (0.48%)	0.37 (0.65%)	0.02 (0.03%)	1.52 (0.96%)
Artocarpus ovatus Blanco	0.01 (0)	0 (0)	0 (0)	0 (0)	0.01 (0.02%)	0 (0)
Astronia cumingiana S.Vidal	0.28 (0.09%)	0 (0)	0.02 (0.06%)	0.05 (0.09%)	0.14 (0.24%)	0.05 (0.03%)
Barringtonia racemosa Spreng.	1.55 (0.49%)	0 (0)	0.09 (0.25%)	0.20 (0.36%)	0.42 (0.70%)	0.84 (0.53%)
Bischofia javanica Blume	15.86 (4.97%)	0 (0)	1.02 (2.74%)	4.38 (7.83%)	2.86 (4.77%)	7.60 (4.79%)
Breynia rhamnoides Müll.Arg.	0.29 (0.09%)	0 (0)	0.17 (0.46%)	0.08 (0.14%)	0.04 (0.07%)	0 (0)
Bridelia penangiana Hook.f.Bridelia insulana Hance	1.05 (0.33%)	0 (0)	0.06 (0.16%)	0.01 (0.02%)	0 (0.01%)	0.98 (0.62%)
Callicarpa elegans Hayek	0.01 (0)	0 (0)	0 (0)	0 (0)	0.01 (0.02%)	0 (0)
Calophyllum blancoi Planch. & Triana	13.53 (4.24%)	0 (0)	0.24 (0.64%)	0.84 (1.50%)	3.43 (5.72%)	9.02 (5.69%)
Calophyllum lancifolium Elmer.	1.39 (0.43%)	1.35 (8.08%)	0.01 (0.03%)	0 (0)	0.02 (0.04%)	0 (0)
Cananga odorata (Lam.) Hook. f. & Thomson	0.06 (0.02%)	0 (0)	0.06 (0.15%)	0 (0)	0 (0)	0 (0)
Canarium calophyllum Perkins.	0.81 (0.25%)	0.01 (0.06%)	0.11 (0.30%)	0.21 (0.37%)	0.13 (0.22%)	0.36 (0.23%)
Canarium hirsutum	0.09 (0.03%)	0 (0)	0 (0)	0.03 (0.05%)	0.05 (0.08%)	0.02 (0.01%)
Canarium luzonicum (Blume) A.Gray	0.62 (0.19%)	0 (0)	0.04 (0.11%)	0.03 (0.05%)	0.12 (0.20%)	0.43 (0.27%)
Canthium fenicis (Merr.) Merr.	0.31 (0.10%)	0 (0)	0.17 (0.47%)	0 (0)	0.13 (0.22%)	0 (0)
Canthium monstrosum (A. Rich.) Merr.	0.16 (0.05%)	0 (0)	0.15 (0.40%)	0.01 (0.02%)	0 (0)	0 (0)
Carallia brachiata (Lour.) Merr.	0.20 (0.06%)	0 (0)	0.06 (0.15%)	0.01 (0.02%)	0.04 (0.07%)	0.09 (0.06%)
Caryota cumingii Lodd. ex Mart.	3.03 (0.95%)	0 (0)	0.87 (2.37%)	0.43 (0.77%)	0.45 (0.74%)	1.29 (0.81%)
Casuarina equisetifolia L.	0.18 (0.06%)	0.18 (1.06%)	0 (0)	0.01 (0.01%)	0 (0)	0 (0)
Celtis philippensis Blanco	0.78 (0.25%)	0 (0)	0 (0)	0 (0)	0.02 (0.03%)	0.77 (0.48%)
Cinnamomum cebuense Kostermans	1.12 (0.35%)	0 (0)	0.05 (0.13%)	0.98 (1.75%)	0.09 (0.15%)	0 (0)
Cleistanthus venosus C.B. Rob.	0.02 (0.01%)	0 (0)	0.01 (0.02%)	0 (0)	0.02 (0.03%)	0 (0)
Cocos nucifera L.	0.02 (0.01%)	0 (0)	0 (0)	0 (0)	0.02 (0.04%)	0 (0)

Cratoxylum celebicum Bl.	1.24 (0.39%)	0 (0)	0.07 (0.18%)	0.01 (0.02%)	1.06 (1.77%)	0.10 (0.06%)
Cycas circinalis L.	0.06 (0.02%)	0 (0)	0 (0)	0.06 (0.10%)	0 (0)	0 (0)
Dendrocnide stimulans (L. f) Chew	0 (0)	0 (0)	0 (0)	0 (0)	0 (0.01%)	0 (0)
Dillenia indica L.	0.02 (0.01%)	0 (0)	0.01 (0.02%)	0 (0)	0.01 (0.02%)	0 (0)
Dillenia philippinensis Rolfe	0.52 (0.16%)	0 (0)	0.02 (0.06%)	0.06 (0.10%)	0.22 (0.37%)	0.22 (0.14%)
Diospyros blancoi A.DC.	1.67 (0.52%)	0 (0)	0.01 (0.02%)	1.64 (2.93%)	0 (0)	0.03 (0.02%)
Diospyros pilosanthera Blanco	1.69 (0.53%)	0 (0)	0.01 (0.02%)	1.04 (1.86%)	0.53 (0.89%)	0.11 (0.07%)
Diplodiscus paniculatus Turcz.	2.86 (0.90%)	0 (0)	1.24 (3.36%)	0.23 (0.41%)	0.81 (1.34%)	0.58 (0.36%)
Dipterocarpus eurynchus Miq.	0.03 (0.01%)	0 (0)	0 (0)	0.03 (0.05%)	0 (0)	0 (0)
Dracontomelon dao (Blanco) Merr. & Rolfe	1.13 (0.36%)	0 (0)	0.01 (0.02%)	0.61 (1.08%)	0.04 (0.07%)	0.48 (0.30%)
Dracontomelon edule (Blanco) Skeels.	0.36 (0.11%)	0 (0)	0 (0)	0.02 (0.04%)	0.02 (0.03%)	0.31 (0.20%)
Dysoxylum cumingianum C. DC.	2.01 (0.63%)	0 (0)	0.15 (0.39%)	0.21 (0.37%)	0.16 (0.26%)	1.50 (0.95%)
Dysoxylum decandrum Merrill.	1.89 (0.59%)	0 (0)	0.01 (0.01%)	0.07 (0.13%)	0.16 (0.27%)	1.65 (1.04%)
Ficus ampelas Burm. f	1.11 (0.35%)	0 (0)	0.13 (0.35%)	0 (0.01%)	0 (0)	0.98 (0.62%)
Ficus balete Merr.	16.97 (5.32%)	0 (0.01%)	0.53 (1.43%)	2.75 (4.91%)	0 (0)	13.69 (8.64%)
Ficus callosa Willd.	0.03 (0.01%)	0 (0)	0 (0)	0.03 (0.05%)	0 (0.01%)	0 (0)
Ficus gul K. Schum. & Lauterb.	1.03 (0.32%)	0.01 (0.03%)	0.47 (1.25%)	0.13 (0.24%)	0.27 (0.45%)	0.15 (0.10%)
Ficus irisana Elmer.	0.21 (0.06%)	0 (0)	0.07 (0.20%)	0.10 (0.18%)	0.03 (0.05%)	0 (0)
Ficus magnoliifolia Blume	0.58 (0.18%)	0 (0)	0.53 (1.44%)	0.04 (0.08%)	0.01 (0.01%)	0 (0)
Ficus minahassae (Teijsm. & De Vriese) Miq.	1.72 (0.54%)	0.01 (0.04%)	0.50 (1.34%)	1.09 (1.94%)	0.02 (0.03%)	0.11 (0.07%)
Ficus nota Merr.	0.36 (0.11%)	0 (0.01%)	0.24 (0.63%)	0.05 (0.09%)	0.01 (0.01%)	0.06 (0.04%)
Ficus odorata (Blanco) Merr.	0.11 (0.04%)	0 (0)	0.09 (0.25%)	0 (0.01%)	0.02 (0.03%)	0 (0)
Ficus septica Burm. f.	0.56 (0.18%)	0 (0.03%)	0.49 (1.33%)	0.07 (0.12%)	0 (0)	0 (0)
Ficus ulmifolia Lam.	0.12 (0.04%)	0 (0)	0.04 (0.12%)	0 (0.01%)	0.06 (0.10%)	0 (0)
Ficus vrieseana Miq.	0.15 (0.05%)	0 (0)	0 (0)	0 (0)	0 (0)	0.15 (0.09%)
Glochidion album (Blanco) Boerl.	0.26 (0.08%)	0 (0)	0.02 (0.06%)	0 (0.01%)	0.01 (0.02%)	0.22 (0.14%)
Glochidion camiguinense Merr.	1.07 (0.34%)	0 (0)	0.22 (0.59%)	0.02 (0.04%)	0.09 (0.16%)	0.74 (0.47%)
Gymnostoma rumphianum (Miq.) L.A.S. Johnson	1.18 (0.37%)	0 (0)	0.38 (1.02%)	0 (0)	0.81 (1.34%)	0 (0)
Heterospathe elata Scheff.	0.34 (0.11%)	0 (0)	0.03 (0.09%)	0.03 (0.05%)	0 (0.01%)	0.28 (0.18%)
Hopea malibato	0 (0)	0 (0)	0 (0)	0 (0)	0 (0.01%)	0 (0)
Hopea philippinensis Dyer	3.12 (0.98%)	0 (0)	0.43 (1.16%)	0.22 (0.40%)	0.08 (0.13%)	2.40 (1.51%)
Horsfieldia costulata (Miq.) Warb.	2.81 (0.88%)	0 (0)	0.34 (0.91%)	0.68 (1.22%)	0.50 (0.83%)	1.29 (0.82%)
Kibatalia gitingensis (Elmer) Woodson	1.87 (0.58%)	0 (0)	0.01 (0.02%)	0.74 (1.33%)	0.89 (1.47%)	0.23 (0.15%)
Knema mindanaensis (Warb.) comb. nov.	0.56 (0.18%)	0 (0)	0.07 (0.18%)	0.02 (0.03%)	0.48 (0.80%)	0 (0)
Leea aculeata Bl.	0.05 (0.02%)	0 (0)	0 (0.01%)	0.02 (0.03%)	0 (0)	0.04 (0.02%)
Leucaena leucaephala (Lam.) de Wit.	0.16 (0.05%)	0 (0)	0 (0)	0.15 (0.27%)	0.01 (0.02) %	0 (0)

Leucosyke capitellata (Pair.) Wedd.	0.69 (0.22%)	0 (0.03%)	0.30 (0.81%)	0.32 (0.57%)	0.02 (0.03%)	0.05 (0.03%)
Lithocarpus llanosii (A.DC.) Rehder	20.40 (6.39%)	0.60 (3.56%)	9.60 (25.87%)	4.62 (8.26%)	4.84 (8.06%)	0.75 (0.48%)
Litsea perrottetii (Bl.) Villar.	0.38 (0.12%)	0 (0)	0.11 (0.30%)	0.08 (0.14%)	0.18 (0.30%)	0.01 (0.01%)
Macaranga bicolor Muell. -Arg.	0.19 (0.06%)	0 (0)	0.14 (0.37%)	0 (0)	0.04 (0.06%)	0.02 (0.01%)
Macaranga tanarius (L.) Müll.Arg.	0.81 (0.25%)	0 (0)	0.63 (1.69%)	0.04 (0.07%)	0.04 (0.06%)	0.10 (0.06%)
Mallotus philippensis (Lam.) Müll.Arg.	0.03 (0.01%)	0 (0)	0.03 (0.08%)	0 (0)	0 (0)	0 (0)
Mallotus ricinoides (Pers.) Müll.Arg.	0.31 (0.10%)	0 (0.01%)	0.06 (0.16%)	0.15 (0.27%)	0.10 (0.17%)	0 (0)
Melicope triphylla (Lam.) Merr.	0.09 (0.03%)	0 (0)	0.02 (0.04%)	0.03 (0.06%)	0.04 (0.06%)	0 (0)
Mussaenda philippica A.Rich.	0.01 (0)	0 (0.02%)	0.01 (0.02%)	0 (0)	0 (0)	0 (0)
Myrica javanica Reinw. ex Bl.	0.35 (0.11%)	0.30 (1.79%)	0 (0)	0 (0)	0 (0)	0.05 (0.03%)
Neolitsea vidalii Merr.	6.08 (1.90%)	0 (0)	0 (0)	2.61 (4.67%)	0.73 (1.22%)	2.73 (1.72%)
Neonauclea bartlingii (DC.) Merr.	0.38 (0.12%)	0 (0)	0 (0.01%)	0.05 (0.09%)	0.17 (0.28%)	0.17 (0.11%)
Neonauclea formicaria (Elmer) Merr.	1.34 (0.42%)	0 (0)	0.17 (0.46%)	0.81 (1.45%)	0.20 (0.34%)	0.16 (0.10%)
Neotrewia cumingii (Müll.Arg.) Pax & K.Hoffm.	0.49 (0.15%)	0 (0)	0.02 (0.06%)	0.01 (0.02%)	0.18 (0.30%)	0.27 (0.17%)
Nephelium lappaceum	0.01 (0)	0 (0)	0 (0)	0.01 (0.01%)	0 (0)	0 (0)
Octomeles sumatrana Miq.	0.25 (0.08%)	0 (0)	0 (0)	0 (0)	0.25 (0.42%)	0 (0)
Ormosia calavensis Blanco	1.53 (0.48%)	0 (0)	0.81 (2.19%)	0.03 (0.05%)	0.66 (1.10%)	0.04 (0.02%)
Palaquium luzoniense (Fern.-Vill.) Vidal	9.60 (3.01%)	0 (0)	0.63 (1.69%)	1.27 (2.27%)	3.32 (5.52%)	4.39 (2.77%)
Pandanus radicans Blanco	0.01 (0)	0 (0)	0 (0)	0 (0)	0.01 (0.02%)	0 (0)
Parartocarpus venenosus (Zoll. & Morr.) Becc. ssp. papuanus (Becc.) Jarr.	0.17 (0.05%)	0.02 (0.15%)	0.02 (0.05%)	0.03 (0.05%)	0.02 (0.04%)	0.08 (0.05%)
Parashorea malaanonan (Blanco) Merr.	106.08 (33.23%)	3.63 (21.73%)	2.54 (6.84%)	13.57 (24.28%)	12.95 (21.59%)	73.38 (46.30%)
Petersianthus quadrialatus (Merr.) Merr.	8.72 (2.73%)	0 (0)	0 (0)	0 (0)	0.39 (0.65%)	8.33 (5.26%)
Piper anduncum L.	0.02 (0.01%)	0.00 (0.03%)	0 (0)	0.00 (0.01%)	0.00 (0.01%)	0.01 (0.01%)
Pipturus arborescens (Link) C. B. Rob.	0.27 (0.08%)	0 (0)	0 (0)	0.08 (0.14%)	0.19 (0.32%)	0 (0)
Planchonella nitida (Blume) Dubard.	1.09 (0.34%)	0 (0)	0 (0)	0.03 (0.06%)	1.06 (1.77%)	0 (0)
Polyalthia flava	2.59 (0.81%)	0 (0)	0.07 (0.19%)	0.01 (0.03%)	0.01 (0.02%)	2.49 (1.57%)
Polyalthia oblongifolia Burck	0.37 (0.12%)	0 (0)	0.17 (0.47%)	0.01 (0.01%)	0.18 (0.31%)	0 (0)
Polyscias nodosa (Blume) Seem.	3.23 (1.01%)	0.04 (0.22%)	0.65(1.75%)	0.82 (1.47%)	1.22 (2.04%)	0.51 (0.32%)
Premna cumingiana Schauer	0.07 (0.02%)	0.00 (0.03%)	0 (0)	0 (0)	0 (0)	0.06 (0.04%)
Premna odorata Blanco	0.14 (0.04%)	0.13 (0.75%)	0 (0)	0.01 (0.03%)	0 (0)	0 (0)
Premna stellate Merr.	0.04 (0.01%)	0 (0)	0 (0)	0.04 (0.07%)	0 (0)	0 (0)
Pterocarpus indicus Willd.	1.51 (0.47%)	1.51 (9.01%)	0 (0)	0 (0)	0 (0)	0 (0)
Pterospermum diversifolium Bl.	0.02 (0.01%)	0 (0)	0 (0)	0.01 (0.02%)	0 (0)	0 (0)
Pterospermum obliquum Blanco	0.01 (0)	0 (0)	0 (0)	0 (0)	0 (0)	0 (0)
Radermachera pinnate (Blanco) Seem.	2.83 (0.89%)	0.01 (0.03%)	1.21 (3.25%)	0.87 (1.56%)	0.58 (0.97%)	0.17 (0.11%)

Securinega flexuosa (Muell. -Arg.)	0.45 (0.14%)	0 (0)	0.16 (0.42%)	0.15 (0.27%)	0.14 (0.23%)	0 (0)
Senna siamea (Lam.) Irwin et Barneby	0.02 (0)	0 (0)	0 (0)	0.01 (0.02%)	0.01 (0.01%)	0 (0)
Shorea almon Foxw.	0.40 (0.13%)	0 (0)	0.01 (0.02%)	0 (0)	0.40 (0.66%)	0 (0)
Shorea contorta Vidal	12.41 (3.89%)	0.26 (1.57%)	2.35 (6.32%)	6.59 (11.80%)	2.67 (4.45%)	0.53 (0.34%)
Shorea guiso (Blanco) Blume	8.24 (2.58%)	0 (0)	2.57 (6.92%)	0.71 (1.27%)	0.20 (0.34%)	4.76 (3.01%)
Shorea palosapis (Blanco) Merr.	2.47 (0.77%)	0 (0)	0.55 (1.49%)	0.35 (0.63%)	0.03 (0.05%)	1.54 (0.97%)
Shorea polysperma (Blanco) Merr.	10.36 (3.25%)	7.39 (44.23%)	0.61 (1.65%)	2.19 (3.91%)	8.17 (13.62%)	1.02 (0.64%)
Strombosia philippinensis (Baill.) Rolfe	1.61 (0.50%)	0 (0)	0.25 (0.67%)	0.54 (0.97%)	0.20 (0.34%)	0.62 (0.39%)
Syzygium crassilimbum (Merr.) Merr.	0.09 (0.03%)	0 (0)	0.03 (0.07%)	0.02 (0.04%)	0.04 (0.06%)	0 (0)
Syzygium gigantifolium (Merr.) Merr.	0.62 (0.19%)	0 (0)	0.22 (0.59%)	0 (0)	0.29 (0.48%)	0.11 (0.07%)
Syzygium hutchinsonii (C.B.Robinson) Merr.	1.13 (0.36%)	0.06 (0.34%)	0.16 (0.44%)	0.23 (0.42%)	0.47 (0.79%)	0.21 (0.13%)
Syzygium surigaense (Merr.) Merr.	2.51 (0.79%)	0 (0)	0 (0)	0 (0)	0.05 (0.09%)	2.45 (1.55%)
Tectona grandis L. f.	0.14 (0.04%)	0 (0)	0 (0)	0 (0)	0.14 (0.24%)	0 (0)
Terminalia microcarpa Decne.	0.03 (0.01%)	0 (0)	0 (0)	0.03 (0.05%)	0 (0)	0 (0)
Toona philippinensis Elmer.	0.84 (0.26%)	0 (0)	0.01 (0.01%)	0.13 (0.23%)	0.13 (0.21%)	0.58 (0.36%)
Trema orientalis (L.) Bl.	0.08 (0.03%)	0.00 (0.01%)	0 (0)	0 (0)	0 (0)	0.08 (0.05%)
Trichospermum involucratum (Merr.) Elmer	0 (0)	0 (0)	0 (0)	0 (0)	0 (0)	0 (0)
Vitex quinata (Lour.) F. N. Williams	0 (0)	0 (0)	0 (0)	0 (0)	0 (0)	0 (0)
Vitex turczaninowii (Turcz.) Merr.	0 (0)	0 (0)	0 (0)	0 (0)	0 (0)	0 (0)
Wrightia pubescens R.Br.	0.80 (0.25%)	0 (0)	0.56 (1.50%)	0.12 (0.21%)	0.13 (0.22%)	0 (0)
Xanthostemon verdugonianus Naves	1.19 (0.37%)	1.19 (7.10%)	0 (0)	0 (0)	0 (0)	0 (0)
Anungo	0.98 (0.31%)	0 (0)	0.17 (0.46%)	0.04 (0.07%)	0.03 (0.04%)	0.75 (0.47%)
Nandamai	0.001 (0.001%)	0 (0)	0 (0)	0 (0)	0.01 (0.01%)	0 (0)
Pandukaki	0.003 (0.001%)	0 (0)	0 (0)	0 (0)	0.003 (0.01%)	0 (0)
Pegonngon	0 (0)	0 (0)	0 (0)	0 (0)	0.003 (0.01%)	0 (0)
Poelig	0.80 (0.25%)	0 (0)	0.56 (1.5%)	0.12 (0.21%)	0.13 (0.22%)	0 (0)
Siyao	1.19 (0.37%)	1.19 (7.10%)	0 (0)	0 (0)	0 (0)	0 (0)

* Values in the parenthesis indicatse the percentage share of respective species in sites total living woody biomass carbon.

Supplementary Table 2. Site environmental attributes, mean±SE. Where, *EL* = elevation, *SL* = slope, *PS* = patch size, *DIS* = distance (from the nearest control forest site), *LAI* = leaf area index, *SOC* = soil organic carbon.

**Site attributes (unit)**	**Fallow category**				**old-growth forest**
	≤5 year	6-10 year	11-20 year	21-30 year	
EL (masl)	600.8 (±22.19)	549.0 (±72.41)	567.2 (±49.24)	574.8 (±35.35)	512.4 (±54.77)
SL (degree)	33 (±5.7)	32.4 (±9.4)	32.6 (±9.2)	38.2 (±7.98)	36.4 (±9.71)
PS (ha)	1.16 (±0.21)	1.14 (±0.13)	1.34 (±0.24)	1.14 (±0.22)	na
DIS (m)	290 (±74.16)	540 (±114.01)	162 (±198.17)	256 (±153.88)	na
LAI (%)	1.33 (±0.57)	5.70 (±0.96)	5.14 (±1.01)	5.33 (±0.69)	6.08 (±0.86)
SOC (%)	6.17 (±0.68)	6.54 (±2.05)	5.21 (±0.69)	6.79 (±1.92)	4.77 (±1.11)

Values in the parenthesis indicates the standarard deviation;

Supplementary Table 3. Wood density and characteristics of species recorded from the sites on Leyte Island, the Philippines.

**Botanical name**	**Local name**	**Origin[1]**	**Successional guild[2]**	**Wood density[3] (gm cm^{-3})**
Alangium javanicum (Bl.) Wang	Putian	Native	Secondary	0.78
Dracontomelon dao (Blanco) Merr. & Rolfe	Dao	Native	Secondary	0.63
Dracontomelon edule (Blanco) Skeels.	Lamio	Native	Secondary	0.55
Cananga odorata (Lam.) Hook. f. & Thomson	Ilang-ilang	Native	Secondary	0.35
Polyalthia oblongifolia Burck	Lapnisan	Native	Secondary	0.56
Polyalthia flava	Yellow lanutan	Endemic	Secondary	0.51
Alstonia macrophylla G. Don	Batino	Native	Pioneer	0.56
Alstonia parvifolia Merr.	Batino-liitan	Endemic	Pioneer	0.46
Wrightia pubescens R.Br.	Lanete	Native	Pioneer	0.53
Kibatalia gitingensis (Elmer) Woodson	Laneteng-gubat	Endemic	Secondary	0.46
Arthrophyllum cenabrei Merr.	Bingliu	Endemic	Pioneer	0.41
Polyscias nodosa (Blume) Seem.	Malapapaya	Native	Pioneer	0.37
Areca cathecu L	Bunga	Native	Secondary	0.62
Cocos nucifera L.	Coconut	Native	Pioneer	0.62
Caryota cumingii Lodd. ex Mart.	Pugahan	Native	Pioneer	0.62
Heterospathe elata Scheff.	Sagisi	Native	Pioneer	0.62
Radermachera pinnate (Blanco) Seem.	Banai-Banai	Native	Secondary	0.52
Canarium hirsutum	Milipili	Native	Secondary	0.57
Canarium calophyllum Perkins.	Pagsahingin-bulog	Native	Secondary	0.57
Canarium luzonicum (Blume) A.Gray	Piling-liitan	Endemic	Pioneer	0.43
Calophyllum lancifolium Elmer.	Bitanghol-sibat	Native	Secondary	0.61
Trema orientalis (L.) Bl.	Anabiong	Native	Pioneer	0.36
Celtis philippensis Blanco	Malaikmo	Native	Secondary	0.72
Casuarina equisetifolia L.	Agoho	Exotic	Pioneer	0.92
Gymnostoma rumphianum (Miq.) L.A.S. Johnson	Mountain agoho	Native	Secondary	1.0
Calophyllum blancoi Planch. & Triana	Bitanghol	Native	Secondary	0.53
Terminalia microcarpa Decne.	Kalumpit	Native	Secondary	0.57
Cycas circinalis L.	Pitogo	Native	Pioneer	0.57
Octomeles sumatrana Miq.	Binuang	Native	Secondary	0.32
Dillenia indica L.	Handapara	Native	Secondary	0.65
Dillenia philippinensis Rolfe	Katmon	Endemic	Secondary	0.67
Shorea almon Foxw.	Almon	Endemic	Climax	0.44
Parashorea malaanonan (Blanco) Merr.	Bagtikan	Native	Climax	0.47
Dipterocarpus eurynchus Miq.	Basilanapitong	Endemic	Secondary	0.72
Hopea philippinensis Dyer	Gisok-gisok	Endemic	Pioneer	0.75
Shorea guiso (Blanco) Blume	Guijo	Native	Pioneer	0.77
Shorea palosapis (Blanco) Merr.	Mayapis	Endemic	Climax	0.42
Anisoptera thurifera	Palosapis	Native	Secondary	0.65
Shorea polysperma (Blanco) Merr.	Tangile	Endemic	Climax	0.56
Shorea contorta Vidal	White lauan	Endemic	Climax	0.48
Hopea malibato	Yakal-kaliot	Native	Climax	1.0
Diospyros pilosanthera Blanco	Bolong-eta	Native	Secondary	0.75
Diospyros blancoi A.DC.	Kamagong	Native	Climax	0.9
Neotrewia cumingii (Müll.Arg.) Pax &	Apanang	Native	Pioneer	0.55

K.Hoffm.				
Mallotus philippensis (Lam.) Müll.Arg.	Banato	Native	Secondary	0.68
Macaranga tanarius (L.) Müll.Arg.	Binunga	Native	Pioneer	0.48
Macaranga bicolor Muell. -Arg.	Hamindang	Endemic	Pioneer	0.30
Mallotus ricinoides (Pers.) Müll.Arg.	Hinlaumo	Native	Pioneer	0.42
Ormosia calavensis Blanco	Bahai	Endemic	Climax	0.50
Albizia falcataria (L.) Fosberg.	Falcata	Exotic	Secondary	0.31
Leucaena leucaephala (Lam.) de Wit.	Ipil-ipil	Exotic	Pioneer	0.64
Pterocarpus indicus Willd.	Narra	Native	Secondary	0.74
Albizia saponaria(Lour.) Blume ex Miq.	Salingkugi	Native	Secondary	0.66
Senna siamea (Lam.) Irwin et Barneby	Thailand shower	Native	Secondary	0.68
Lithocarpus llanosii (A.DC.) Rehder	Ulaian	Native	Pioneer	0.71
Cratoxylum celebicum Bl.	Paguriagon	Native	Secondary	0.60
Vitex quinata (Lour.) F. N. Williams	Kalipapa	Native	Pioneer	0.50
Vitex turczaninowii (Turcz.) Merr.	Lingo-lingo	Endemic	Secondary	0.64
Premna cumingiana Schauer	Magilik	Native	Secondary	0.66
Tectona grandis L. f.	Teak	Exotic	Pioneer	0.61
Callicarpa elegans Hayek	Tigau-ganda	Endemic	Pioneer	0.40
Cinnamomum cebuense Kostermans	Kaningag	Endemic	Pioneer	0.50
Litsea perrottetii (Bl.) Villar.	Marang	Native	Secondary	0.49
Neolitsea vidalii Merr.	Puso-puso	Endemic	Secondary	0.59
Barringtonia racemosa Spreng.	Putat	Native	Pioneer	0.50
Petersianthus quadrialatus (Merr.) Merr.	Toog	Endemic	Pioneer	0.59
Diplodiscus paniculatus Turcz.	Balobo	Endemic	Secondary	0.63
Pterospermum obliquum Blanco	Kulatingan	Endemic	Pioneer	0.52
Astronia cumingiana S.Vidal	Badling	Native	Pioneer	0.56
Dysoxylum decandrum Merrill.	Igyo	Native	Pioneer	0.58
Toona philippinensis Elmer.	Lanigpa	Native	Pioneer	0.42
Dysoxylum cumingianum C. DC.	Tara-tara	Native	Pioneer	0.64
Artocarpus blancoi (Elmer) Merr.	Antipolo	Endemic	Pioneer	0.60
Artocarpus ovatus Blanco	Anubing	Endemic	Pioneer	0.69
Ficus irisana Elmer.	Aplas	Native	Pioneer	0.44
Ficus balete Merr.	Balete	Native	Pioneer	0.65
Ficus gul K. Schum. & Lauterb.	Butli	Native	Secondary	0.44
Ficus minahassae (Teijsm. & De Vriese) Miq.	Hagimit	Native	Secondary	0.38
Ficus septica Burm. f.	Hauili	Native	Secondary	0.44
Ficus ulmifolia Lam.	Is-is	Endemic	Pioneer	0.44
Ficus callosa Willd.	Kalukoi	Native	Pioneer	0.33
Ficus magnoliifolia Blume	Kanapai	Native	Secondary	0.44
Ficus odorata (Blanco) Merr.	Pakiling	Endemic	Climax	0.44
Ficus vrieseana Miq.	Tagitig	Native	Secondary	0.44
Ficus nota Merr.	Tibig	Native	Pioneer	0.44
Ficus ampelas Burm. f	Upling-gubat	Native	Pioneer	0.33
Myrica javanica Reinw. ex Bl.	Hindang	Native	Secondary	0.62
Knema mindanaensis (Warb.) comb. nov.	Bunod	Endemic	Secondary	0.58
Parartocarpus venenosus (Zoll. & Morr.) Becc. ssp. papuanus (Becc.) Jarr.	Malanangka	Native	Pioneer	0.42
Horsfieldia costulata (Miq.) Warb.	Yabnob	Native	Pioneer	0.49
Ardisia pyramidalis (Cav.) Pers. ex A. DC.	Aunasin	Native	Secondary	0.58
Syzygium surigaense (Merr.) Merr.	Kagagko	Endemic	Secondary	0.71
Syzygium crassilimbum (Merr.) Merr.	Kaitatanag	Endemic	Secondary	0.71
Syzygium gigantifolium (Merr.) Merr.	Malatalisai	Endemic	Secondary	0.71

Syzygium hutchinsonii (C.B.Robinson) Merr.	Malatambis	Endemic	Secondary	0.71
Xanthostemon verdugonianus Naves	Mangkono	Endemic	Pioneer	1.05
Strombosia philippinensis (Baill.) Rolfe	Tamayuan	Endemic	Secondary	0.75
Pandanus radicans Blanco	Ulangong-ugatan	Endemic	Pioneer	0.33
Securinega flexuosa (Muell. -Arg.)	Anislag	Endemic	Pioneer	0.77
Antidesma ghaesembilla Gaertn.	Binayuyu	Native	Climax	0.63
Glochidion camiguinense Merr.	Bunot-Bunot	Endemic	Pioneer	0.61
Glochidion album (Blanco) Boerl.	Malabagang	Native	Pioneer	0.61
Breynia rhamnoides Müll.Arg.	Matang-hipon	Native	Pioneer	0.77
Cleistanthus venosus C.B. Rob.	Sarimisim	Endemic	Secondary	0.67
Bridelia penangiana Hook.f.Bridelia insulana Hance	Subiang	Native	Secondary	0.54
Bischofia javanica Blume	Tuai	Native	Secondary	0.64
Piper anduncum L.	Spiked pepper	Native	Pioneer	0.39
Carallia brachiata (Lour.) Merr.	Bakauan-gubat	Native	Secondary	0.70
Neonauclea formicaria (Elmer) Merr.	Hambabalud	Endemic	Pioneer	0.74
Mussaenda philippica A.Rich.	Kahoi-dalaga	Native	Secondary	0.64
Neonauclea bartlingii (DC.) Merr.	Lisak	Endemic	Secondary	0.74
Canthium fenicis (Merr.) Merr.	Mapugahan	Endemic	Pioneer	0.64
Canthium monstrosum (A. Rich.) Merr.	Tadiang-anuang	Native	Pioneer	0.43
Melicope triphylla (Lam.) Merr.	Matang-arau	Endemic	Pioneer	0.38
Nephelium lappaceum	Rambutan	Native	Pioneer	0.77
Planchonella nitida (Blume) Dubard.	Duklitan	Exotic	Secondary	0.61
Palaquium luzoniense (Fern.-Vill.) Vidal	Nato	Endemic	Secondary	0.71
Pterospermum diversifolium Bl.	Bayok	Native	Pioneer	0.58
Trichospermum involucratum (Merr.) Elmer	Langosig	Endemic	Pioneer	0.33
Leucosyke capitellata (Pair.) Wedd.	Alagasi	Native	Pioneer	0.44
Pipturus arborescens (Link) C. B. Rob.	Dalunot	Native	Pioneer	0.39
Dendrocnide stimulans (L. f) Chew	Lingaton	Native	Pioneer	0.21
Premna odorata Blanco	Alagau	Native	Secondary	0.66
Premna stellate Merr.	Manaba	Native	Secondary	0.66
Leea aculeata Bl.	Amamali	Native	Pioneer	0.57
Unknown	Anungo	-	Secondary	0.57
Unknown	Nandamai	-	Pioneer	0.57
Unknown	Pandukaki	-	Pioneer	0.57
Unknown	Pegonngon	-	Secondary	0.57
Unknown	Poelig	-	Pioneer	0.57
Unknown	Siyao	-	Pioneer	0.57

[1]where Native – refers to a species naturally occurring in the Philippines, Endemic - refers to a species found only in the Philippines and Exotic – refers to a species that has been introduced in the Philippines.

[2]based on experts opinion from the Philippines; where pioneer species generally refers to the early colonizer species with high light demanding and first growing nature, secondary are the intermediary group of species with moderate light demand and growth, and climax species are the characteristics species of a forest ecosystem that are common in the old growth forest, and are shade tolerant.

[1]meadian wood density value retrieved from the World Agroforestry Centre Wood Density Database on 2014 (http://www.worldagroforestry.org/regions/southeast_asia/resources/db/wd).

Supplementary Table 4. Organic carbon contents in litter and undergrowth samples from the study sites on Leyte Island, the Philippines.

**Site category**	**Carbon (%) in dry biomass***	
	Litter	Undergrowth
SA0-5	39.56 (±2.52)	37.74 (±3.91)
SA6-10	41.17 (±5.49)	39.53 (±3.19)
SA11-20	41.46 (±2.13)	44.04 (±1.85)
SA21-30	43.45 (±2.57)	44.14 (±1.10)
SF	40.37 (±1.73)	42.57 (±1.70)

*values in the parenthesis indicate the standard deviation of means.

Supplementary Table 5. Pearson correlation between site environmental attributes.

	Fallow age	***Elevation***	***Slope***	***Patch size***	***Distance***	***LAI***	***SOC***
Fallow age	1	-0.09	0.25	0.07	-0.35	0.57**	0.05
Elevation	-0.09	1	0.52*	-0.003	-0.12	-0.41	0.53*
Slope	0.25	0.52*	1	-0.16	0.07	0.02	0.43
Patch size	0.07	-0.003	-0.16	1	-0.09	-0.04	-0.33
Distance	-0.35	-0.12	0.07	-0.09	1	0.22	-0.01
LAI	0.57**	-0.41	0.02	-0.04	0.22	1	-0.01
SOC	0.05	0.53*	0.43	-0.33	-0.01	-0.01	1

*Correlations significant at $p<0.05$ level;

**Correlations significant at $p<0.01$ level.
